# Insights into Synonymous Codon Usage Bias in Hepatitis C Virus and Its Adaptation to Hosts

**DOI:** 10.3390/pathogens12020325

**Published:** 2023-02-15

**Authors:** Rekha Khandia, Azmat Ali Khan, Noushad Karuvantevida, Pankaj Gurjar, Igor Vladimirovich Rzhepakovsky, Isabel Legaz

**Affiliations:** 1 Department of Biochemistry and Genetics, Barkatullah University, Bhopal 462026, India; 2 Pharmaceutical Biotechnology Laboratory, Department of Pharmaceutical Chemistry, College of Pharmacy, King Saud University, Riyadh 11451, Saudi Arabia; 3 College of Medicine, Mohammed Bin Rashid University of Medicine and Health Sciences, Dubai P.O. Box 505055, United Arab Emirates; 4 Department of Science and Engineering, Novel Global Community Educational Foundation, Hebersham, NSW 2770, Australia; 5 Medical and Biological Faculty, North Caucasus Federal University, 355017 Stavropol, Russia; 6 Department of Legal and Forensic Medicine, Biomedical Research Institute (IMIB), Regional Campus of International Excellence “Campus Mare Nostrum”, Faculty of Medicine, University of Murcia, 30120 Murcia, Spain

**Keywords:** hepatitis C virus (HCV), codon usage, liver cirrhosis, hepatocellular carcinoma, similarity index, relative codon deoptimization index, allograft rejection, liver transplantation

## Abstract

Hepatitis C virus (HCV) is enveloped RNA virus, encoding for a polyprotein that is processed by cellular proteases. The virus is responsible for liver cirrhosis, allograft rejection, and human hepatocellular carcinoma. Based on studies including compositional analysis, odds ratio analysis, parity analysis, skew analysis, relative synonymous codon usage, codon bias, and protein properties, it was evident that codon usage bias in HCV is dependent upon the nucleotide composition. Codon context analysis revealed CTC-CTG as a preferred codon pair. While CGA and CGT codons were rare, none of the codons were rare in HCV-like viruses envisaged in the present study. Many of the preferred codon pairs were valine amino acid-initiated, which possibly infers viral infectivity; hence the role of selection forces appears to act on the HCV genome, which was further validated by neutrality analysis where selection accounted for 87.28%, while mutation accounted for 12.72% force shaping codon usage. Furthermore, codon usage was correlated with the length of the genome. HCV viruses prefer valine-initiated codon pairs, while HCV-like viruses prefer alanine-initiated codon pairs. The HCV host range is very narrow and is confined to only humans and chimpanzees. Based on indices including codon usage correlation analysis, similarity index, and relative codon deoptimization index, it is evident in the study that the chimpanzee is the primary host of the virus. The present study helped elucidate the preferred host for HCV. The information presented in the study paved the way for generating an attenuated vaccine candidate through viral recoding, with finely tuned nucleotide composition and a perfect balance of preferred and rare codons.

## 1. Introduction

The Hepatitis C virus (HCV) is an enveloped, single-stranded RNA virus of 9.6 kb genome size flanked by 5′ and 3′ untranslated regions. A single polyprotein is transcribed of over 3000 amino acids cleaved into structural and nonstructural proteins [1]. HCV is a major cause of liver cirrhosis and hepatocellular carcinoma, with increased mortality and dismal survival worldwide [2,3]. Liver cancer may be prevented using new curative hepatitis C antivirals [4]. Liver transplantation in HCV-positive recipients displays higher mortality rates, and HCV infection leads to allograft rejection [5]. Understanding the virus-host interactions can help prevent and control HCV infection. The HCV displays a limited host range where it robustly infects only two hosts, humans and chimpanzees [6].

Degeneracy in the genetic code allows the encoding 20 amino acids from 61 codons. Except for TAA, TAG, and TGA, which encode for stop codon, and Met and Trp, which are encoded by only a single codon, all other amino acids are encoded by multiple codons, known as synonymous codons. However, synonymous codons are not randomly used, and specific codons are preferred over others, called codon bias. Codon bias can be tissue-specific [7], organ-specific [8], species-specific [9], and environment-dependent [10].

Since the viruses are intracellular pathogens, they rely on the host machinery to replicate and exhibit various levels of selection while infecting different hosts. Co-evolution and adaptation of the viruses to the hosts are the widely studied parameters that are analyzed using synonymous codon usage bias [11]. Codon bias is the result of non-random mutational patterns, selectional forces, and genome composition. It is related to the gene expression level [12], gene length [13], selective transcription [14], presence of rare codons [15], preferred codons [16], preferred codon pairs [17], protein properties [18], mRNA structure [19] roles of translation efficacy [20] and accuracy [21]; accordingly, virus fitness in any host [22] may be explained. Unfit viruses are attenuated in their infectivity and may serve as a vaccine candidate.

In the present study, we envisaged 54 genomes of the HCV virus (complete polyprotein) and various parameters like compositional parameters, nucleotide disproportion, dinucleotide frequency, codon usage, codon bias, relative synonymous codon usage, presence of rare codons, preferred codon pairs, the effect of major evolutionary forces, and gene expression were studied. The relative codon deoptimization index (RCDI) and similarity index analysis was carried out for its hosts, chimpanzee and human, to determine which host the virus is more adapted to. The analysis helps in understanding molecular signatures and the extent of mutational and selectional forces associated with HCV, and also provide information that can be useful in designing a vaccine candidate against it.

## 2. Materials and Methods

### 2.1. Sequence Collection

The complete coding sequences for the hepatitis C virus (HCV) were retrieved from National Centre for Biotechnology Information (http://www.ncbi.nlm.nih.gov, Accessed on 10 November 2022). Based on the criteria for selecting the sequence, the polyprotein sequence must be complete, devoid of ambiguous nucleotides, divisible by three, and start with the start codon and terminate with the stop codon; a total of 64 sequences were qualified. Out of these 64 sequences, 54 belonged to 1a, 1b, 2a, 2b, 2c, 2k, (non-recombinants) while 3a, 5a, and 6a genotypes were present as recombinant with 2a genotype in ten recombinant isolates. The information on accession numbers, genotypes, and resistance status towards antivirals are given in the supplementary file S1. To rationally compare the data from HCV with data from other HCV-like viruses, we selected 03 other HCV-like viruses Bovine hepacivirus (BovHepV), Equine hepacivirus (EqHV) and rodent hepacivirus (RHV). For BovHepV, EqHV, and RHV, 27, 23, and 41 sequences were used to compare with HCV. These HCV-like sequences were also retrieved from the NCBI database, and the selection criteria were kept the same as for HCV.

### 2.2. Base Composition

The overall base composition (A%, T%, G%, and C%) and the base composition at the third codon position (A3%, T3%, G3%, and C3%) were analyzed. Average %GC content with GC content at all three codon positions (GC1%, GC2%, and GC3%) was also determined.

### 2.3. Dinucleotide Odds Ratio

There may be 16 dinucleotides derived from 4 bases, influencing codon bias and amino acid composition [23]. The odds ratio is the likelihood of observing a dinucleotide and is calculated using the formula online available link https://www.bioinformatics.nl/cgi-bin/emboss/compseq, accessed on 15 November 2022). Here, the values below 0.78 and above 1.23 are considered underrepresentation and overrepresentation of dinucleotide, respectively [24].

### 2.4. Nucleotide Skew

A disproportionate usage of nucleotide is termed nucleotide skew, and it originates from the asymmetric replication in replication fork, mutational and selectional biases [25]. Nucleotide skews, namely, AT skew, GC skew, purine skew, pyrimidine skew, amino skew, and keto skew, were calculated using the formula Skew = (A + B) / (A − B) where A and B are former and later nucleotides [26]. A positive skew value indicates the abundance of the first nucleotide over the second one, and vice versa [27].

### 2.5. Codon Usage

Codon usage analysis for HCV and its hosts, humans (*Homo sapiens*) and chimpanzees (*Pan troglodytes*), and HCV like viruses and their respective hosts *Bos taurus* (bovine), *Eqqus caballus* (equine), and *Peromyscus maniculatus* (rodent), for BovHepV, EqHV, and RHV was done using the Kazusa database (https://www.kazusa.or.jp/codon/, accessed on 10 November 2022). The codon usage by each organism is given in the supplementary file S2. The codon usage here describes as the usage of codon per thousand codons. The codon usage values (per thousand) obtained from the Kazusa database was based on 93487 and 857 CDSs for human and chimpanzee genomes, respectively. HCV, BovHepV, EqHV and RHVHV codon usage per thousand was calculated using CAICal software [28].

### 2.6. Relative Synonymous Codon Usage (RSCU)

RSCU is a statistical tool to determine the codon usage bias of a single gene or entire genome. It is observed to the expected frequency of codon out of many synonymous codons available for a single amino acid in a given gene, set of genes, or genome [29]. RSCU values above 1.6 suggest overrepresentation, while values below 0.6 show underrepresentation [30,31]. RSCU values were obtained by the CAICal server (https://www.kazusa.or.jp/codon/, accessed on 10 November 2022) developed by Puigbo P et al. (2008) [28].

### 2.7. Neutrality Plot Analysis

A neutrality plot is a method to determine the influence of two major evolutionary forces, selection and mutation, on the gene. The neutrality plot is constructed by regressing %GC12 at the Y axis and %GC3 at the X axis, and it accounts for the mutation-selection equilibrium during codon bias. Each of the genes is represented as a dot in the plot. A regression coefficient value less than 0.5 suggests a more significant role of natural selection, while greater than 0.5 suggests a greater impact of mutational pressure [32].

### 2.8. The Effective Number of Codons (ENc)

The effective number of codons (ENc) is one of the common measures to explain the usage bias of synonymous codons [33]. It ranges between 20 to 61. When only one codon is used out of many available synonymous codons, a value of 20 is achieved and indicates the highest bias. On the other hand, when all the synonymous codons are used equally, a value of 61 is obtained, which is suggestive of no bias. Generally, values above 35 are considered low bias [13]. The ENc values were calculated using the COUSIN tool (https://cousin.ird.fr/, Accessed on 15 November 2022) [34].

### 2.9. Codon Adaptation Index (CAI)

The CAI is the similarity measure between the synonymous codon usage of a gene and that of a reference set. It may be used as a predictive of the protein expression level of genes [35]. Higher CAI values indicate higher adaptability and a higher expression level, and indicate the codons with higher RSCU values [36]. A web-based server CAIcal (http://genomes.urv.cat/CAIcal/, accessed on 11 November 2022) [28] was used to calculate CAI using the reference set of *Homo sapiens* and *Pan troglodytes* to determine the expression level of HCV in these two host organisms. The CAI value ranges between 0 and 1. For BovHepV, EqHV and RHV the reference set of *Bos taurus*, *Equus caballus* and *Peromyscus maniculatus* was used.

### 2.10. Parity Rule 2 (PR2) Bias Plot

A PR2 bias plot is suggestive of the comparative magnitude of mutation and selection forces acting together on the composition of a gene or genome. If the nucleobases are distributed proportionately across the plot, it indicates the influence of mutational force, while disproportionately distributed data points indicate the role of selection and mutation [32]. Here GC and AT bias at the third codon position is evaluated by plotting the GC bias [G3 / (G3 + C3)] on the X axis and the AT bias [A3 / (A3 + T3)] on the Y axis. If A and T and G and C bases are equal, it will result in a value of 0.5, and there is no bias between mutation and selection [37].

### 2.11. Codon Pair Context and Preferred Codons

Codon context is a preference of codon pairs in a given sequence. It is often linked to the accuracy of the translation [38] and speed [39]. The 5′ codon context, rare codon and genome comparison analysis was performed using the ANACONDA^®^ 2software (https://docs.anaconda.com/anaconda/install/hashes/win-2-64/, accessed on 10 November 2022) [38].

### 2.12. Protein Properties

General Average Hydropathicity (GRAVY) and Aromaticity (AROMA) are the frequency of hydrophobic and aromatic amino acids in a protein, and influence shape codon usage bias [40]. A positive GRAVY value indicates the dominance of hydrophobic amino acids, while a negative value suggests hydrophilic amino acids [41]. The correlation of these protein properties with GC%, GC3%, Nc, and CAI indicates the effect of selection force on codon usage bias [42].

### 2.13. Similarity Index Analysis

Similarity index analysis identifies the effect of host codon usage patterns on shaping codon usage of the pathogen. The similarity index was calculated by the formula given by Zhou and colleagues [43]. The values near Zero suggest a high similarity in codon usage between the host and pathogen, and values near 1 indicate a significant divergence [44].

### 2.14. The Relative Codon Deoptimization Index (RCDI) Analysis

The relative codon deoptimization index (RCDI) was calculated for the polyproteins of 64 HCV strains for chimpanzees and human hosts [45]. RCDI compares the similarities between the given gene set and the reference set and provides an idea about the rate of viral gene translation in the host. Values closer to 1 show higher translation rates and a more host-adapted codon usage pattern [30], while values higher than 1 show deoptimization in the codon usage pattern of the pathogen with that of a host. RCDI was calculated with *Pan troglodytes* and *Homo sapiens* as reference sequences using the RCDI/eRCDI server (http://genomes.urv.cat/CAIcal/RCDI/, accessed on 14 November 2022) [46].

### 2.15. Principal Component Analysis

The principal component analysis (PCA) is a multivariate analysis to analyze the major trends present between the variables. The PCA analysis of RSCU values of 64 coding sequences of HCV was done using Origin18 statistical software (https://www.originlab.com/index.aspx?go=Support&pid=3301, accessed on 10 November 2022). RSCU values of each codon were distributed into 59 vectors, each corresponding to one codon (excluding Met, Trp, and stop codons). Here, RSCU values were converted into uncorrelated variables.

### 2.16. Phylogenetic Tree Construction

Based on the RSCU values, a phylogenetic tree was constructed to evaluate the relatedness among the HCV sequences using Ward’s cluster analysis method. Past4 program (https://www.nhm.uio.no/english/research/resources/past/, Paleontological Statistics software version 4.03, accessed on 10 November 2022) was used for clustering, and the figure was generated in Mega10 software [47].

## 3. Results

### 3.1. Compositional Analysis

Compositional analysis revealed that the HCV genome is GC rich. The %A and %T composition was 20.18 ± 0.37% and 21.39 ± 0.27%, respectively, while %C and %G were 30.12 ± 0.84% and 28.29 ± 0.27%, respectively. Overall %GC composition was 58.42 ± 1.05% and %AT was 41.57 ± 1.05%. A similar trend of the richness of C and G nucleotides was also observed at the third codon position, where %A3 and %T3 had 14.02 ± 1.06% and 18.21 ± 1.9% compositions, respectively. In contrast, for %C3 and %G3, it is 38.23 ± 2.0% and 29.52 ± 0.85% respectively. Comparative analysis of overall %GC composition and %GC composition at all the three codon positions reveals that among the three codon positions, %GC3 content was highest with an average value of 67.76 ± 2.7% while %GC2 was least with an average value of 50.57 ± 0.31%.

**Figure 1:**
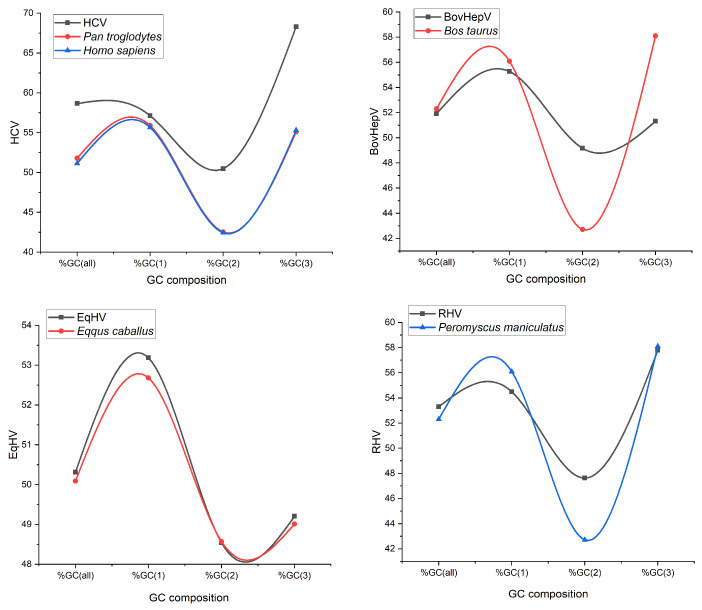
GC composition analysis for HCV and HCV like viruses with their respective hosts.

Similar to HCV, in HCV-like viruses also, the sequences are GC-rich. Overall GC content is 51.92%, 50.31%, and 53.30% for BovHepV, EqHV, and RHV, respectively. A comparison of overall GC content at different codon positions was done for the HCV and envisaged HCV-like viruses. It revealed that overall, %GC is higher in HCV, RHVHV and EqHV compared to their respective hosts, except BovHepV. Furthermore, the percent GC2 is higher than the host’s %GC2 in all viruses except for EqHV, which is the same for the virus and host (Figure 1).

### 3.2. Odds Ratio Analysis Revealed Overrepresentation of GpG and CpCwhile Underrepresentation of TpA, ApA, TpT, and ApT

**Figure 2:**
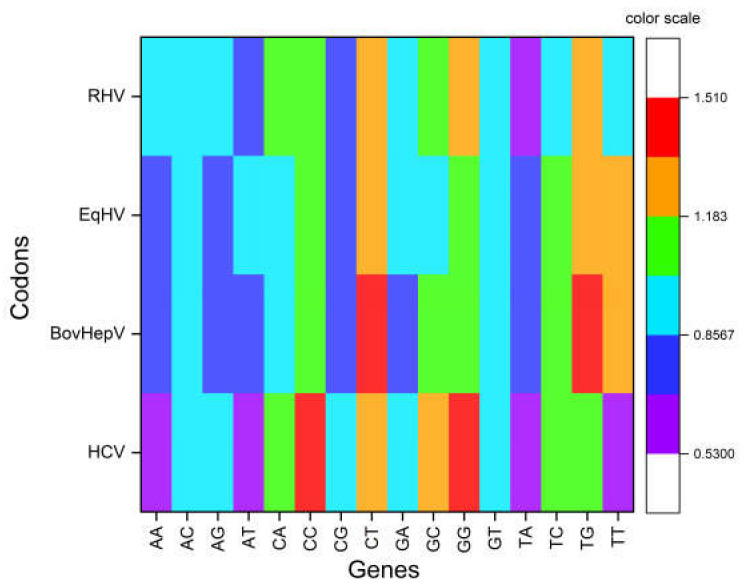
Dinucleotide odds ratio for HCV and HCV like viruses.

Dinucleotide odds ratio analysis indicated that four dinucleotides, TpA, ApA, TpT, and ApT were underrepresented (Odds ratio < 0.78) while two dinucleotides, GpG and CpC, were overrepresented (odds ratio > 1.23). CpG dinucleotide was randomly used (odds ratio 0.98) in HCV genomes. The odds ratio was compared with other HCV-like viruses’ odds ratio, and the underrepresentation of TpA, ApA, TpT, and ApT was found to be a peculiar feature of HCV (Figure 2).

### 3.3. Selection Force Is Dominant Force in the Shaping Codon Usage

A neutrality plot between %GC3 and %GC12 is used to determine the level at which mutation and selection forces influence codon usage in any sequence. If there is a correlation between %GC12 and %GC3, it is suggestive of the role of mutation force in influencing codon bias at all three codon positions [48]. In the present study, we found a positive correlation between %GC12 and %GC3 (*r* = 0.791, *p* < 0.0001) in HCV. Relative neutrality was 12.72%, while selective constraint was 87.28%, suggested the prominent role of selection force in modeling codon bias in HCV polyprotein sequences. At the same time correlation between %GC12 and %GC3 indicates the role of mutational force. Therefore, it can be inferred that both the selection and mutational forces act on HCV polyprotein sequences. The R2 value of 0.5957 indicated that 59.57% variation in %GC12 is attributed to the %GC3 composition.

In BovHepV, we found a native correlation, however insignificant, between %GC12 and %GC3 (*r* = −0.296, *p* = 0.13). Relative neutrality was 7.21%, while selective constraint was 92.79%. R2 value 0. 0881% suggested that 8.81% variation in %GC12 is due to %GC3.

For EqHV, no correlation between %GC12 and %GC3 was found. Relative neutrality was 6.69%, while selective constraint was 93.31%, suggestive of selection as the dominant force determining the sequence of EqHV. The R2 value 0.0603 suggested 6.03% variations in %GC12 is attributed to the %GC3 composition.

**Figure 3:**
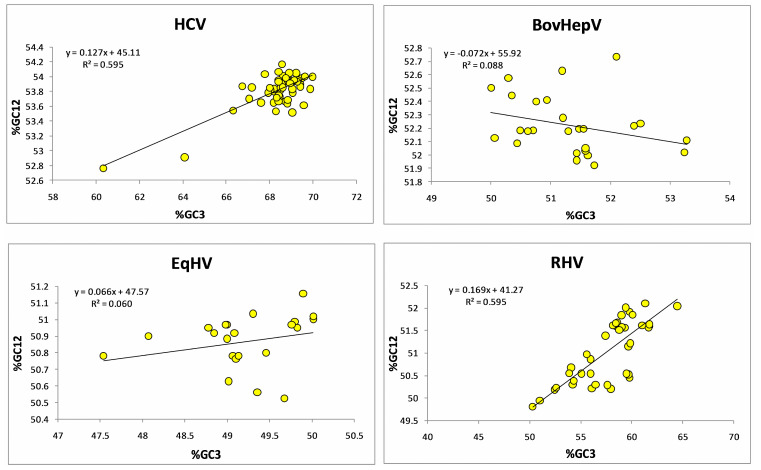
Neutrality plot analysis for HCV and HCV-like viruses suggests the dominant role of selection force in shaping codon usage.

For RHV, a positive correlation (*r* = 0.771, *p* < 0.0001) between %GC3 and %GC12 has been observed. The relative neutrality of 16.93% and selective constraints of 83.07% suggested a dominant role in selection. The R2 value 0.5952 suggested 59.52% variation in %GC12 is attributed to the %GC3 composition. Overall analysis suggested a dominant role of selective forces acting on HCV and HCV-like viruses influencing codon usage [49] (Figure 3).

### 3.4. Parity Plot Analysis Indicated Dominance of Pyrimidine over Purines

**Figure 4:**
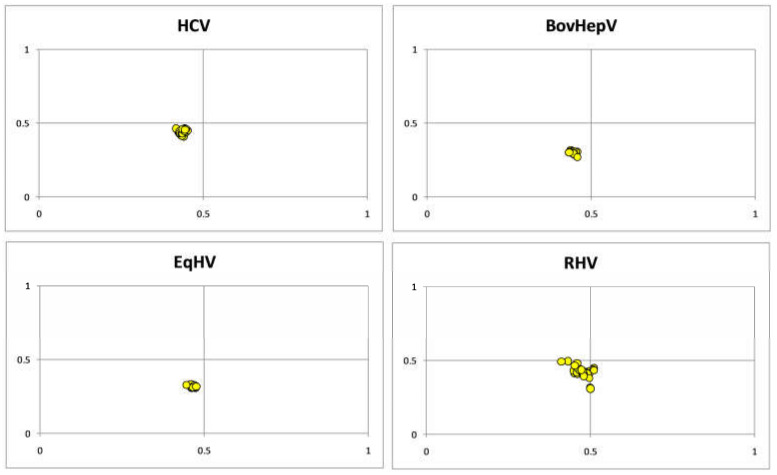
Parity plot for HCV and HCV like-viruses revealed preference of C and T over G and A.

The parity plot is constructed by plotting GC bias (G3 / G3 + C3) at X-axis and AT bias (A3 / A3 + T3) at Y axis [50]. For HCV, the value of GC bias was 0.434 ± 0.007, and AT bias was 0.433 ± 0.013. GC bias was 0.443 ± 0.007, 0.464 ± 0.007, 0.475 ± 0.021 while AT bias was 0.297 ± 0.009, 0.318 ± 0.008, 0.421 ± 0.039 for BovHepV, EqHV and RHV, respectively. It concludes that nucleotides C and T are preferred over G and A at the third codon position and suggest selection force [51] in HCV and HCV-like viruses (Figure 4).

### 3.5. Result of Skew

Skew is a disproportionate usage of the nucleotide. We performed the correlation analysis for skews with the gene expression and codon bias. Skew values were calculated for the 64 polyprotein sequences of HCV and were correlated with CAI and ENc. AT skew and GC skews are generally used to determine the compositional distributions of nucleotides [52]. In the present study, the mean AT and GC skew values were −0.028 and −0.033, respectively and it suggested that T and C are preferred over A and G. The average values of other skews were also negative (−0.170, −0.175, −0.202 and −0.143 for purine, pyrimidine, amino and keto skews, respectively). An index related to gene expression CAI is shown to be influenced by nucleotide skew [53]. Except for AT skew, all the skews were significantly negatively correlated with gene expression (*p* < 0.001). ENc, a non-directional codon usage bias measure, was positively correlated with all the skews except for AT skew (*p* < 0.001). A significant correlation between codon bias and skew is found in Nipah virus also [54].

### 3.6. RSCU Analysis Revealed the Overrepresentation of G/C Ending Codons

RSCU analysis revealed that eight codons ending with G/C were overrepresented in the genes. TCC, ATC, CTC, AGG, CTG, ACC, CCC, and GGC were eight overexpressed codons (RSCU > 1.6) [55] in 100%, 96.29%, 96.29%, 92.59%, 85.18%, 70.37%, 57.4%, and 61.11% of HCV polyprotein sequences. On the other hand, thirteen A/T ending codons, TTA, CTA, ATT, GTT, GTA, AGT, AAT, GAT, GAA, TGT, CGT, CGA, and GGA, showed underrepresentation in 100%, 87.03%, 81.48%, 64.81%, 100%, 98.14%, 64.81%, 81.48%, 87.03%, 57.41%, 51.85%, 92.59%, and 72.22% of HCV genomes.

In HCV, out of 18 synonymous codons, all the 18 preferred codons were GC ending. In BovHepV, 07 GC ending and 11 AT-ending, in EqHV, 05 GC ending codons while 13 AT-ending, and in RHVHepV, 17 GC ending and 01 AT-ending codon were present. The results suggest different codon usage patterns in HCV and HCV-like viruses.

### 3.7. Protein Properties Are Dependent on the Composition and Codon Bias

We did correlation analysis for the nucleotide composition at all three codon positions. GRAVY and AROMA are the protein features that showed a positive, negative or no correlation. GRAVY showed a significant positive correlation with GC composition at all codon positions, while AROMA had no correlation (Table 1). Overall, the analysis revealed that composition significantly influences the hydropathicity and aromaticity of polyproteins encoded by the HCV genome [56].

Hydropathicity and aromaticity are the protein properties that are thought to influence mRNA transcripts, thereby affecting codon bias [57]. Pearson correlation analysis revealed that codon bias index ENc was negatively correlated with the GRAVY and AROMA (*r* = −0.581, *p* < 0.001; *r* = −0.281, *p* < 0.05, respectively). The results suggest that the codon bias decreases with a decrease in the frequency of hydrophobic and aromatic amino acids. PCs are representative of dinucleotide and codon composition. Thus, we investigated the correlation of protein properties with PCs and found that GRAVY positively correlated with both PC1 and PC2 (*r* = 0.633, *p* < 0.001 and *r* = 0.322, *p* < 0.05). In contrast, AROMA showed a positive correlation with PC1 (*r* = 0.553, *p* < 0.001) and a negative correlation with PC2 (*r* = −0.275, *p* < 0.05). The correlation of GRAVY and AROMA with ENc and PCs showed that protein properties influence codon usage.

### 3.8. ENc Indicated Low Bias

ENc is the index to point to codon usage bias. Fifty-four HCV polyproteins ranged from 56.46 to 50.68, averaging 51.92 ± 0.88. ENc values above 50 in the present study show weaker bias [58]. ENc values ranged between 56.07 to 54.43 (average 55.37 ± 0.36), 55.83 to 54.49 (average 55.25 ± 0.41), and 58.41 to 53.36 (average 56.48 ± 1.08) for BovHepV, EqHV and RHVs respectively. Overall, the analysis indicated a low bias in HCV and HCV-like sequences. Furthermore, the bias showed a negative correlation with the gene lengths (*r* = −0.280, *p* < 0.05) and is suggestive that the bias increases with the increase in the length of the HCV polyprotein sequence [59]. Contrary to HCV, RHV showed a positive correlation between ENc and length; thus, bias decreases with the increase in length in the case of RHV. In the case of both the BobHepV and EqHV, no correlation was observed between codon bias and length (*r* = 0.351, *p* = 0.11 for EqHV and *r* = 0.041, *p* = 0.83 for BovHepV).

### 3.9. Codon Context Analysis Revealed an Abundance of CTC-CTG Codon Pair and Rarity of CGA and TTA

Variations in the codon contexts in the top 20 codon pairs were determined for HCV. The trend of the favored codon pair is depicted in Figure 2. The HCV genome (polyprotein segment) had both the preferred (depicted with green color) and rejected (depicted with red color) codon pairs. CTC-CTG codon pair was most abundant, and six codon pairs were initiated with Val amino acid (GTC/GTG) in native HCVs. At the same time, the GCC-CTC pair was most abundant in recombinant HCVs, with no such preference in the codon pair initiation. GCT-GCT, GGC-GCT, and again the GCT-GCT codon pairs were most abundant in BovHepV, EqHV, and RHVHepV, with a maximum of six, five, and four alanine-initiated codons in BoVHepV, EqHV, and RHV, respectively. The top 20 codon pairs are given in Table 2. Here it is interesting to see that HCV top 20 most occurring codon pairs encompassed six valine-initiated codons; contrarily, all other envisaged three HCV-like viruses have a preference for alanine-initiated codons.

A codon with a frequency of less than 0.5% was considered rare [60]. CGA (Arg) and TTA (Leu) codons were rarely used in the native HCV genome. In recombinant HCVs, GCA (Arg) and CGT (Arg) were rare. On the other hand, in HCV-like viruses like BovHepV, eqHV and RHV, none of the codons have a frequency of less than 0.5% (except for stop codons).

**Figure 5:**
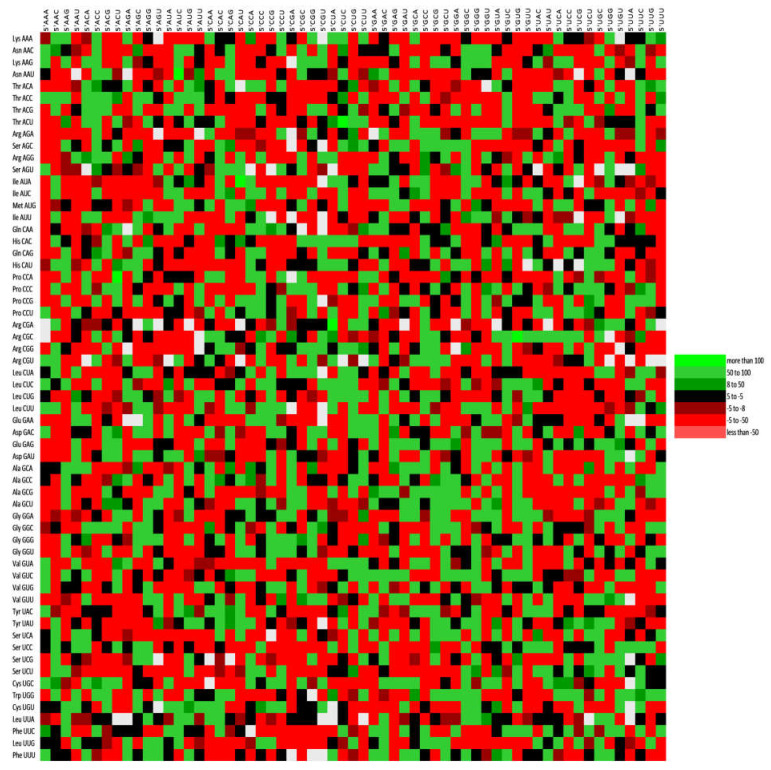
Codon context analysis for 54 polyprotein sequences of HCV. The green color shows a positive context, while red shows a negative context. Black color shows insignificant context, and grey indicates no context.

Codon pair bias was investigated for the HCV virus, and all three kinds of contexts (positive, negative or no bias) were present (Figure 5).

**Figure 6:**
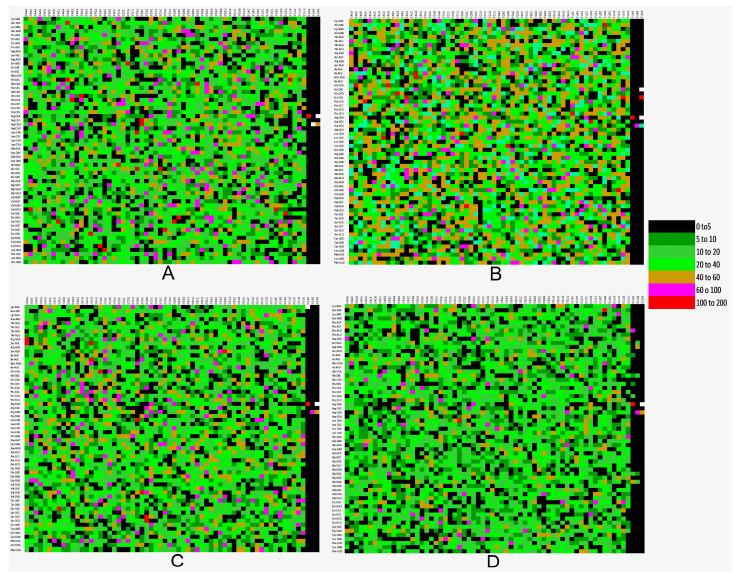
Differential display map for comparison of codon pair bias for genomes (**A**). HCV native and HCV recombinant (**B**). HCV and BovHepV (**C**). HCV and EqHV (**D**). HCV and RHV.

We then investigated the difference in codon context in different virus groups. To display the difference, we constructed a differential display map (DDM) between the sequences from two groups. No context or low context difference is depicted by residual values less than 20, while high context difference is depicted by residual values more than 100 [61] (Figure 6A). Comparison of HCV sequence with BovHepB (Figure 6B), EqHv (Figure 6C) and RHV (Figure 6D) are depicted below. From the results, it is clear that codon context or codon pair differences are prominent.

### 3.10. PCA Analysis

**Figure 7:**
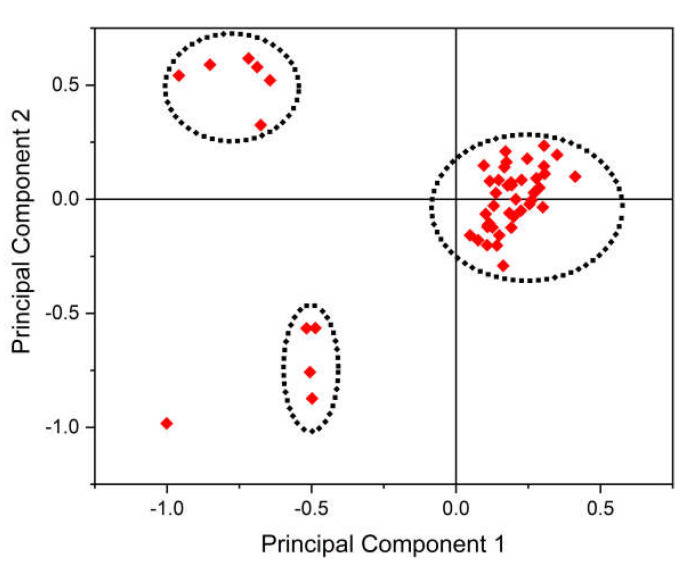
PCA analyses revealed low to moderate codon bias in HCV genomes. Red dots indicate each of the native HCVs. HCV 2k isolate took a different position on PCA.

PCA is often used for reduction in dimensions. RSCU values of 59 synonymous codons were taken as 59 vectors. Most of the data points were clustered at three sites only, except three data points, and it is suggestive that most of the HCV genomes’ codon usage mainly follows three kinds of trends. Only one data point was scattered (HCV 2k), indicating that its codon usage pattern differed from the rest of the HCV genomes. For native HCVs, primary and secondary axis contributed for 28.60%, and 19.84% variation, respectively. Some of the data points were scattered far from the axes, indicating low to moderate bias in codon usage in these genomes [13]. The graph also indicated more bias in a few HCV genomes than others (Figure 7).

### 3.11. Phylogeney Analysis

**Figure 8:**
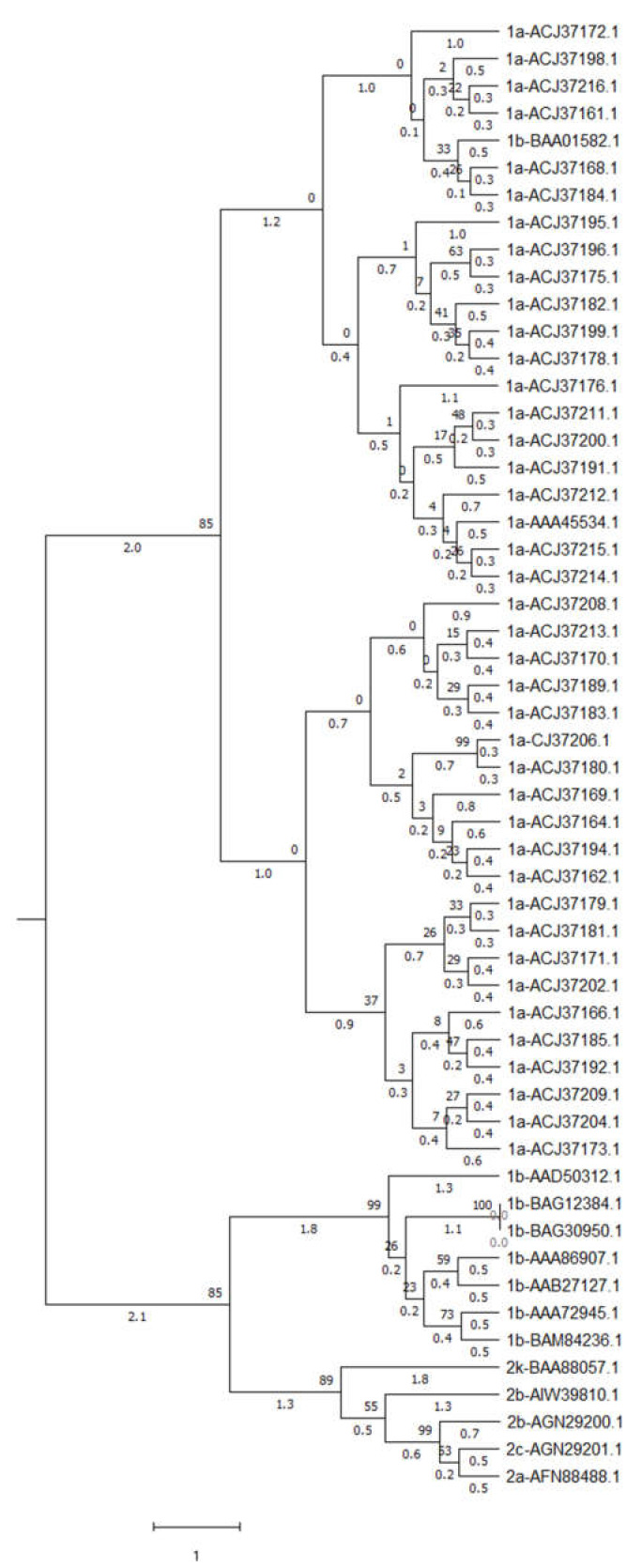
Phylogenetic analysis for 54 polyprotein sequences of HCV reveals that the sequences may be separated into two clusters based on Ward’s hierarchical agglomerative clustering.

Phylogenetic analysis of 54 HCV genomes was carried out using Ward’s hierarchical agglomerative clustering method with a 500-bootstrap value. The analysis revealed that genomes might be separated into two clusters. Fifteen polyprotein sequences formed one cluster, while the rest of the sequences made another cluster. The separation of clusters indicates that each cluster has different codon usage pattern (Figure 8).

### 3.12. Adaptability of HCV Genome for its Hosts Human and Chimpanzee

#### 3.12.1. The Codon Adaptation Index Reveals More Adaptability of HCV for Humans Compared to Chimpanzees

CAI values are used to determine the expression level of a pathogen in a host, or in other words, it indicates the adaptation of a virus into its hosts [36]. The genes or pathogens with higher CAI values in a host are considered more adapted than those with lower CAI values. The obtained CAI values in human and chimpanzee is given in supplementary file S3. For chimpanzees, it ranged between 0.665 to 0.721, while for humans, it ranged between 0.722 to 0.758. The average CAI values were 0.714 ± 0.006 and 0.751 ± 0.004 for chimpanzees and humans. The results indicate that the virus is more adapted to humans than chimpanzees. CAI values were 0.696 ± 0.003, 0.639 ± 0.003, and 0.402 ± 0.017 for BovHepV, EqHV, and RHV, respectively, for their respective hosts. Results suggested that the HCV virus is well adapted to humans and chimpanzees compared to other animal HCV-like viruses in their respective hosts. Adaptation is the least for RHV in its host, *Peromyscus maniculatus*. The CAI results are in concordance with the data of RCDI.

#### 3.12.2. Codon Usage Pattern of HCV Is More Similar with That of Chimpanzee Codon Usage Pattern

We compared the codon usage per thousand for HCV, humans, and chimpanzees; the results are given in Figure 5. In addition, correlation analysis of codon usage between the hosts human and chimpanzee, and pathogen HCV was done to determine to which host the codon usage pattern of HCV matches more. The linear Pearson correlation analysis revealed that codon usage of HCV is near to the codon usage of chimpanzees (*r* = 0.712, *p* < 0.001) than humans (*r* = 0.59, *p* < 0.001) and is suggestive of chimpanzees as the primary host. Similarly, a statistically significant Pearson correlation was present between codon usage of *Bos taurus* and BovHepV (*r* = 0.605, *p* < 0.001), *Equus caballus* and EqHV (*r* = 0, 0.948, *p* < 0.001), and *Peromyscus maniculatus* and RHV (0.648, *p* < 0.001). The higher correlation of codon usage between the host and the pathogen is suggestive of adaptation.

#### 3.12.3. HCV Displays the Highest Codon Usage Deoptimization for Human

The higher similarity of codon usage between host and pathogen is depicted by RCDI values closer to 1 [45]. It can be used to improve protein expression in a heterologous expression system. A lower RCDI is suggestive of more adaptation, and at the same time a higher RCDI also suggests that few of the genes are expressed in latency phases, or maybe the virus is present with a low replication rate. Here the RCDI value was 1.08 ± 0.01 and 1.11 ± 0.01 for chimpanzees and humans, respectively, suggesting that HCV codons are more deoptimized to humans. The RCDI value of 1.19 ± 0.01, 1.19 ± 0.01, and 1.81 ± 0.17 for BovHepV, EqHV and RHV showed that HCV-like viruses BovHepV, and EqHV are better adapted to their respective hosts, *Bos taurus* and *Equuscaballus*, compared to RHV in one of its hosts *Peromyscus maniculatus*. Unfortunately, we could not compare the RCDI of RHV for its more common host *Lophuromys* (*L. dudui, L. machangui, L. stanleyi, L. laticeps*), since the genomic sequences were not available for them.

#### 3.12.4. Similarity Index showed Pan Troglodytes Is Primary Host

The host with a lower similarity index will have more similar codon usage than the host with a higher similarity index [62]. The similarity index was 0.03 and 0.039 for *Pan troglodytes* and *Homo sapiens*, respectively. The results indicate that HCV codon usage similarity is more with *Pan troglodytes* than *Homo sapiens*. The similarity index was 0.066, 0.001, and 0.031 for BovHepV, EqHV and RHV in *Bos taurus*, *Equuscaballus* and *Peromyscus maniculatus*, respectively. Results suggested that EqHV is most adapted to its host *Equus caballus*.

## 4. Discussion

HCV virus causes chronic and fatal hepatic liver problems leading to liver cirrhosis and hepatocellular carcinoma, with increased mortality and dismal survival worldwide [2,3]. In the present study, we performed codon usage analysis, codon pair, and other molecular patterns analysis that helped determine the host and the necessary information that might be useful in generating synthetic biology-based vaccine candidates. Since 2011, many HCV-like viruses have been identified in several hosts, including dogs [63], equine [64], bats [65], cattle [66], rodents [67], and monkeys [68] in a mammalian group. Full-length genome sequences were available: canine 01, equine 26, bat 06, bovine 27, monkey 01, and rodent 41. To add statistically correct controls, we compared HCV polyprotein sequences with qualified HCV-like animal viruses; those are BovHepV (27 sequences), EqHV (23 sequences) and RHV (41 sequences).

In an attempt to see the effects of skew variation, a short stretch of the HIV-I pol region was systematically manipulated by adding and removing A nucleotide. As a result, nucleotide A content was altered from 40.2% (wild type) to increase to 46.9% or reduced to 31.7% and 26.3%. Here AG skew was affected dramatically, and a reduced viral replication has been observed in the virus having maximum nucleotide A content [69]. Hence, it is depicted that skews decide viral fitness also. The skew may be manipulated by using optimized or deoptimized codons or codon pairs while making a vaccine candidate, depending on the type of vaccine candidate.

In each organism, the odds ratio is unique, and CpG dinucleotide bias has been observed in genomes of humans and mice [70] and may be used as a molecular signature. In HCV, the odds ratio analysis indicated the underrepresentation of TpA, ApA, TpT and ApT dinucleotide while GpG and CpC were overrepresented. CpG odds ratio is in normal ranges for large DNA viruses while small DNA viruses have low CpG [71,72]. All papillomaviruses and polyomaviruses are CpG depleted [73]. In case of RNA viruses, generally an underrepresentation of CpG is found, with the exception of rubella virus where CpG dinucleotide is present in normal ranges owing to the exceptionally high GC (up to 70%) content [74]. The dinucleotide odds ratio of a pathogen is also an indicator of host pathogen interactions. The members of Flaviviridae viruses infecting vertebrates exhibit depletion of both the CpG (possibly induced by the methylation-deamination process) and TpA, while those infecting non-vertebrates had only TpA depletion [75]. In the present study, HCV is a RNA virus and in the HCV genome the GC content is high. Thus, it is reflected on the dinucleotide content and the CpG dinucleotide, which is generally otherwise an underrepresented dinucleotide, and is present in normal ranges (between 0.78 to 1.3) here. The normal range CpG odds ratio is likely owing to the high GC content in the HCV genome. Based on nucleotide composition, the overrepresentation of GpG and CpC and the underrepresentation of TpA, ApA, TpT and ApT dinucleotides may be explained. Dinucleotide composition becomes symmetrical for complementary dinucleotide for all of the four underrepresented (TpA/ApT and ApA/TpT) and overrepresented (GpG/CpC) dinucleotides due to the double-stranded nature of DNA, which is here in case of HCV. Interestingly, HCV is an RNA virus, and thus we are unable to explain the reason behind this specific dinucleotide pattern. The high bias towards CpC (and GpG owing to complimentary to the CpC dinucleotide) may also be explained as a fine-tuning process of protein expression [76]. Remarkably, underrepresentation of TpA/ApT and ApA/TpT and overrepresentation of GpG/CpC was a feature of HCV viruses which is absent in HCV like viruses.

Apart from codon bias, codon pair bias also exists, demonstrating the likelihood of two codons’ presence together. Few codon pairs are favored [77,78] while few are avoided [79] than expected in a protein-coding region. In the context of viruses, codon optimization in gag and pol genes of HIV-1 did not improve the viral replication, but optimization results in virus attenuation [80]. However, contrary results were seen by Jordan-Paiz [81] in the HIV-1 envelope gene, where codon pair deoptimization doesn’t necessarily generate attenuation while optimizing attenuated virus replication in MT-4 cells. Hence, the effect of codon pair optimization or deoptimization is gene-specific and can’t be generalized. Codon pair analysis revealed that for HCV, CTC-CTG, while for BovHepV, EqHV, and RHV, GCT-GCT, GGC-GCT and GCT-GCT codon pairs were most preferred. In the present study, it was found that out of the most preferred 20 codon pairs in HCV and HCV-like viruses, HCV had maximum valine amino acid initiated codon pairs. In contrast, other envisaged HCV-like viruses preferred alanine-initiated codon pairs. It might relate to the initiation of protein synthesis with alanine without the involvement of met-tRNA, as present in the Drosophila C virus, Himetobi P virus and Rhopalosiphum padi virus [82].

Since lower RCDI is suggestive of better adaptation to the host [36] and RCDI for *Pan troglodytes* is lower than *Homo sapiens*, it may be inferred that in chimpanzees, the HCV is better adapted than humans. Our results concord with the data obtained with other HCV-like viruses where low RCDI suggested a better adaptation of BovHepV and EqHV in their respective hosts, *Bos taurus* and *Equus caballus* owing to lower RCDI. On the other hand, RHV is less adapted in *Peromyscus maniculatus* compared to different species of *Lophuromys* (*L. dudui*, *L. machangui*, *L. stanleyi*, *L. laticeps*) [83].

CAI is a quantitative measure of the expression level of a gene in the host using a highly expressed gene set as a reference [28]. A comparison of CAI values, which are the indicators of gene expression in the host, revealed that HCV has higher expression in humans (0.75 ± 0.005) than in chimpanzees (0.71 ± 0.009). This observation contradicts the results we obtained based on RCDI, similarity index, and correlation analysis of codon usage between HCV and hosts. This deviation is possibly due to the reason that here for calculating CAI as a reference set, data from only highly expressed genes is taken as a reference which may not necessarily explain the whole codon usage. Thus, based on our observations, we conclude that *Pan troglodytes* may possibly be the primary host for HCV with high codon usage similarities (based on similarity index), less codon deoptimization (low RCDI), and a higher correlation of codon usage of HCV RSCU with *Pan troglodytes*.

The expression of viruses in the different hosts is different, as evidenced by different CAI and RCDI values of the Nipah virus in 10 different host species. One virus may be adapted differently in various host species. Based on the RCDI and CAI analysis, Nipah virus is best adapted in African green monkeys. Codon pattern analysis helps evaluate the clinical outcome of a pathogen infection where high virus adaptation will result in higher replication, increased infectivity, and clinically pathogenic outcomes of the virus [36]. Although viral fitness and virulence are often coupled, sometimes deviations are also observed owing to complex virus–host interactions. For example, an experiment conducted in the vesicular stomatitis virus (VSV) to evaluate the relationship between viral fitness and virulence revealed that overall, there was a positive correlation between the two was present. However, few outliers were also present, with the higher fitness and low virulence and low fitness with no effect in virulence [84].

Similarly, bats are shown to be highly adapted to the Nipah virus, yet avirulent to the host, with that a low calculated fitness, yet high virulence in a ferret model is exhibited. Although, such examples are rare and result from the complex host–pathogen interaction, and are presented as only a few outliers. Still, the codon usage pattern analysis helps determine the host of a virus [36]. Thus, based on our observations, it is clear that codon usage analysis can likely determine the hosts and clinical outcome of infection for a virus.

## Figures and Tables

**Table 1:** Pearson correlation analysis of GRAVY and AROMA with compositional components of HCV virus.

	%A	%A1	%A2	%A3	%T	%T1	%T2	%T3	%C	%C1
GRAVY (r value)	−0.680	−0.118	−0.487	−0.701	−0.384	−0.553	0.201	−0.303	0.617	0.635
*p* value	***	NS	***	***	**	***	NS	*	***	***
AROMA	−0.134	0.310	−0.270	−0.226	−0.175	−0.132	−0.073	−0.153	0.282	−0.001
*p* value	NS	*	*	NS	NS	NS	NS	NS	*	NS
	%C2	%C3	%G	%G1	%G2	%G3	%GC(all)	%GC(1)	%GC(2)	%GC(3)
GRAVY	−0.655	0.650	0.469	−0.110	0.641	0.176	0.602	0.510	0.277	0.592
*p* value	***	***	***	NS	***	NS	***	***	***	***
AROMA	−0.441	0.418	−0.041	−0.345	0.464	−0.282	0.169	−0.187	0.245	0.221
*p* value	***	**	NS	*	***	*	NS	NS	NS	NS

Level of significance *** *p* < 0.001; ** *p* < 0.01; * *p* < 0.05; NS Non significant.

**Table 2:** Top 20 preferred codon pairs in HCV polyprotein sequences in naive HCV, recombinant HCV and HCV-like viruses.

	HCVs (Recombinants)		HCVs (Non- Recombinants)		Bovine Hepacivirus BovHepV		Equine Hepacivirus (EqHV)		Rodent Hepacivirus (RHV)	
S. No.	Codon Pair	Frequency	Codon Pair	Frequency	Codon Pair	Frequency	Codon Pair	Frequency	Codon Pair	Frequency
1	GCC-CTC	88	CTC-CTG	369	GCT-GCT	258	GGC-GCT	160	GCT-GCT	229
2	CCC-CCC	81	GTG-GCC	320	CTT-GAG	153	TGG-GCT	137	GAG-GAG	216
3	GTC-ATC	75	AAC-ACC	295	GTC-ACC	149	GCT-TGG	132	AAG-AAG	190
4	GGC-GCC	71	GCC-ATC	293	CTT-GCT	142	CTT-GCT	130	GCT-GGC	174
5	TAT-GAC	69	GTC-ACC	270	GTC-ACT	135	GCT-TCT	129	GAG-GAC	164
6	GAG-GTC	63	CTC-ACT	270	GTT-GCT	134	GAC-ACT	121	GCT-GCC	163
7	GCG-GCC	62	GTG-TGC	267	GGT-GCT	134	ACT-GGC	112	GCT-GAG	163
8	GTG-GAC	61	CTG-GAC	267	GGC-ACT	133	GAT-GTT	107	GAG-GCT	156
9	GAC-GCC	61	GCT-GCC	263	GCT-GTG	133	TTT-GAC	101	TTT-GAC	154
10	ACC-ATC	60	AAC-TGG	258	GCT-GTT	131	GCT-TTT	101	GTG-GTG	148
11	ACC-ACC	59	GTG-CGC	257	CCT-TAC	127	GCT-GTT	101	TTG-GCT	146
12	TGC-TCC	58	ATC-ACC	255	ACT-GCT	127	TCT-GTT	100	ACT-GGC	144
13	TGC-GGC	58	GTG-GGG	253	TGG-GCT	126	TGT-GGC	98	ACC-AAG	143
14	GAG-GAG	56	ATC-ATG	249	GAT-GTT	123	GCT-GTC	98	TAC-ACC	141
15	TAC-TCC	53	TGG-GCG	248	GGT-GCC	121	ACT-GTC	97	GAC-ACC	134
16	GAC-ATC	53	TAC-GTG	246	GCT-ACT	119	CCT-TAT	95	TGT-GAC	131
17	GGG-TAC	51	GCC-ACC	243	CCT-GCT	116	GGG-GAT	94	GTG-GCC	130
18	TCC-TGG	50	ATC-AAC	236	GCT-GGC	114	ATG-GGC	92	AAG-GAG	130
19	TAC-ATC	50	GTC-ATC	234	GTT-TGG	111	GAG-GAA	91	AAG-AAA	130
20	GGT-GTG	50	CTG-CTG	234	GCT-GTC	111	TAT-GAC	90	GGG-AAG	129

## Data Availability

Available upon request.

## Supplementary Materials

The following supporting information can be downloaded at: https://www.mdpi.com/article/10.3390/pathogens12020325/s1.
